# Development of rapid guidelines: 1. Systematic survey of current practices and methods

**DOI:** 10.1186/s12961-018-0327-8

**Published:** 2018-07-13

**Authors:** Sergio C. Kowalski, Rebecca L. Morgan, Maicon Falavigna, Iván D. Florez, Itziar Etxeandia-Ikobaltzeta, Wojtek Wiercioch, Yuan Zhang, Faria Sakhia, Liudmila Ivanova, Nancy Santesso, Holger J. Schünemann

**Affiliations:** 1 Department of Health Research Methods, Evidence and Impact and Mac GRADE Center, McMaster University Health Sciences Centre, Room 2C16, 1280 Main Street West, Hamilton, ON L8N 4K1 Canada; 2 Department of Internal Medicine, Division of Rheumatology- Universidade Federal do Paraná, R. Gen. Carneiro, 181, Curitiba, PR Brazil; 3 Hospital Moinhos de Vento, Porto Alegre, Brazil; 4 National Institute of Science and Technology for Health Technology Assessment, Federal University of Rio Grande do Sul, Porto Alegre, Brazil; 5 Department of Pediatrics, University of Antioquia, Cra. 51D #62-29, Medellin, 050001 Colombia; 6 Public Health & Preventive Medicine Residency Program (including Family Medicine), McMaster University, 1280 Main Street West, Hamilton, ON L8S 4L8 Canada; 7 Cochrane Canada Center, McMaster University, Health Sciences Centre, Room 2C14, 1280 Main Street West, Hamilton, ON L8S 4K1 Canada; 8 Department of Medicine, McMaster University, Health Sciences Centre, Room 2C14, 1280 Main Street West, Hamilton, ON L8S 4K1 Canada

**Keywords:** Guideline, Emergencies, Methodology, Rapid reviews, Guideline development, Clinical guidelines

## Abstract

**Background:**

Guidelines in the healthcare field generally should contain evidence-based recommendations to inform healthcare decisions. Guidelines often require 2 years or more to develop, but certain circumstances necessitate the development of rapid guidelines (RGs) in a short period of time. Upholding methodological rigor while meeting the reduced development timeframe presents a challenge for developing RGs. Our objective was to review current practices and standards for the development of RGs. This is the first of a series of three articles addressing methodological issues around RGs.

**Methods:**

We conducted a systematic survey of methods manuals and published RGs to identify reasons for the development of RGs. Data sources included existing guideline manuals, published RGs, Trip Medical Database, MEDLINE, EMBASE and communication with guideline developers until February 2018.

**Results:**

We identified 46 guidelines that used a shortened timeframe for their development. Nomenclature describing RGs varied across organisations, wherein the United States Centers for Disease Control and Prevention produced ‘Interim Guidelines’, the National Institute for Health and Care Excellence in the United Kingdom developed ‘Short Clinical Guidelines’, and WHO provided ‘Rapid Advice’. The rationale for RGs included response to emergencies, rapid increases in cases of a condition or disease severity, or new evidence regarding treatment. In general, the methods to assess the quality of evidence, the consensus process and the management of the conflict of interest were not always clear. While we identified another 11 RGs from other institutions, there was no reference to timeframe and reasons for conducting a RG. The three organisations mentioned above provide guidance for the development of RGs.

**Conclusions:**

There is a lack of standardised nomenclature and definitions regarding RGs and there is inconsistency in the methods described in manuals and in RG. It is therefore important that all RGs provide a detailed and transparent description of their methods in order for readers and end-users to be able to assess their quality and validate their findings.

**Electronic supplementary material:**

The online version of this article (10.1186/s12961-018-0327-8) contains supplementary material, which is available to authorized users.

## Background

Guidelines contain recommendations to inform users (e.g. healthcare providers, general population or patients) about the benefits and harms of a specific intervention or situation to achieve the best health outcome. Guidelines differ depending on their purpose, scope and timeframe for development. Typically, guidelines take 2 years to develop due to the several steps required, including identification of important outcomes, identification of evidence, synthesis and presentation of evidence, peer review, and dissemination and implementation, among others [1]. However, certain situations require the development, dissemination and implementation of guidelines within a condensed timeframe such as in response to a public health emergency or urgent humanitarian crisis [2].

Rapid guidelines (RGs) refer to guidelines that report the use of a shortened timeframe for their development. A challenge with developing RGs is maintaining methodological rigor while meeting a reduced development timeframe. Our objective was to review current practices and standards for the development of RGs. This systematic survey is the first in a series of three articles to inform the process and guiding principles for the development of rapid and evidence-based recommendations [3, 4]. The second article in the series reports on results from interviews with RGs developers, and the third presents recommendations for the expansion of the Guideline Development Checklist and tool for RGs [5].

## Methods

To understand the current practices and standards for the development of RGs, we examined the methods and approaches presented in manuals produced by several guideline development organisations and in published RGs. We developed the protocol in April 2013 (Additional file 1: Appendix 1) and then conducted a systematic survey of published RGs and purposively sampled methods manuals from several organisations to describe current practices and standards for the development of RGs. We focused on identifying the rationale for development, methods and approaches used, as well as the overall quality of the guidelines.

### Systematic survey of RGs

#### Search strategy

We utilised several search strategies to identify organisations that developed guidelines using a shortened timeframe. We conducted searches, from database inception through February 2018, in MEDLINE, EMBASE, and Trip Medical Database, which searches the National Guideline Clearinghouse. We used a combination of the following terms for each database: “rapid”, “fast”, “short”, “interim” (Additional file 1: Appendix 2). In addition, we contacted guideline developers and methodologists from key organisations for their input. Finally, we searched the 35 manuals identified by guideline developers, methodologists and references to develop the GIN-McMaster Guideline Development Checklist (Additional file 1: Appendix 3) [5]. Discrepancies in electronic and manual search strategies were resolved by discussion and consensus.

#### Eligibility criteria

The inclusion criteria for data sources considered in this study were (1) methods manuals and publications focusing on the development of guidelines using a shortened timeframe; (2) guidelines described as ‘rapid’ and/or using a shortened timeframe for their development; and (3) guidelines and manuals published in English. We excluded guideline updates, rapid systematic reviews, rapid health technology assessments, rapid review reports and position statements that did not make recommendations.

#### Selection of studies and data abstraction

To identify current practices used for RG development, we selected up to three of the most recent guidelines identifying a shortened timeframe from organisations that did not publish RGs regularly (i.e. less than five published guidelines between 2011 and 2014). From organisations that published more than five guidelines in that period (considered as regularly publishing RGs), all RGs were selected and assessed. Six investigators (RLM, MF, IDF, II, YZ, WW) independently screened titles and abstracts, and the full text of potentially relevant articles was obtained to determine eligibility. We developed and pilot tested a data abstraction form to extract information about the topics of interest. The abstraction form collected information on (1) condition evaluated; (2) guideline group member composition, (3) number of research questions requiring recommendations; (4) number of recommendations; (5) guideline’s timeframe (including time spent in each step and time to completion); (6) evidence review process; (7) quality of evidence assessment procedures; (8) factors considered when formulating recommendations; (9) considerations about costs and stakeholder involvement; and (10) reasons for developing a RG instead of a standard guideline. An additional file shows the information collected in more detail (Additional file 1). For the pilot phase, four investigators (MF, II, YZ, FS) abstracted data from two RGs, namely ‘Osteoporosis: assessing the risk of fragility fracture’ and ‘Rapid advice: Diagnosis, prevention and management of cryptococcal disease in HIV-infected adults, adolescents and children’ [6, 7]. Subsequently, eight investigators (RLM, MF, IDF, II, YZ, FS, SK, LI) independently and in duplicate reviewed eligible studies and abstracted data.

To assess the overall quality of the guideline, five investigators (MF, II, YZ, SK, WW) applied the Appraisal of Guidelines for Research & Evaluation Instrument (AGREE II) in pairs to all RGs identified and agreement between reviewers was calculated [8]. The AGREE II instrument consists of 23 key items organised within six domains followed by two global rating items. Raters assess the credibility of a guideline using a 7-point scale (1 – strongly disagree, 7 – strongly agree) and calculate separate scores ranging from 0 to 100% for each of the six domains. Higher percentages suggest higher credibility.

### Purposive sampling of methods manuals

The search strategy and eligibility criteria used for identifying methods manuals for guideline development have been described previously [5]. The systematic search identified ‘guidelines for guidelines’, guideline method reports and guideline manuals available from guideline development organisations (e.g. international and national agencies, professional societies, etc.), as well as clinical and public health guidelines describing their development process [5]. In addition, we contacted key stakeholders and topic-specific experts to identify relevant manuals. The topic-specific experts have worked in the guidelines for more than 10 years, served on key committees, and attended meetings in the field. Websites of organisations that develop RGs were reviewed to identify methods manuals.

### Data analysis

We present data narratively with descriptive statistics.

## Results

### Rapid guidelines

#### Search results

**Fig. 1:**
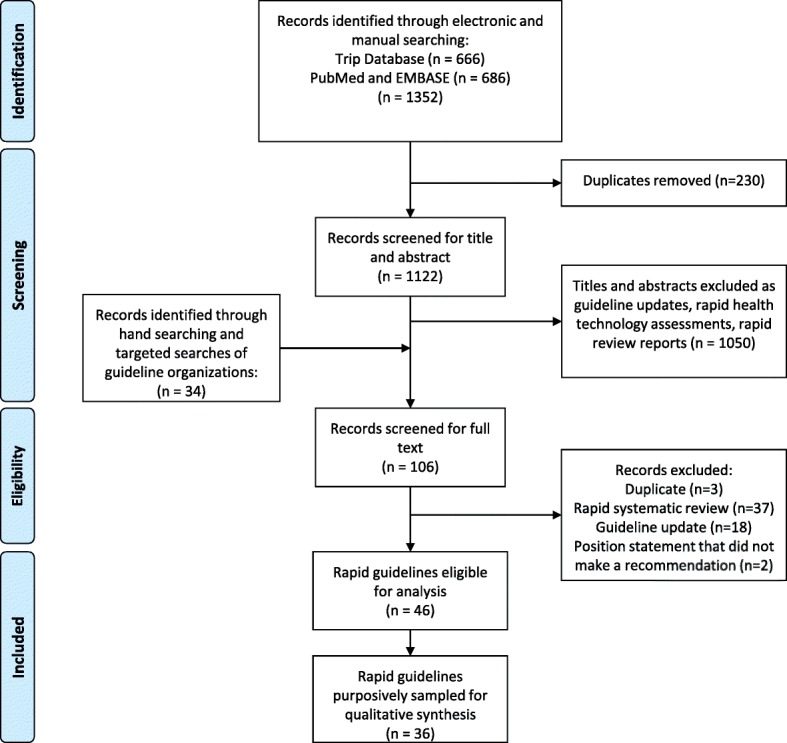
PRISMA flow diagram

We identified 46 guidelines from 11 different organisations using a shortened timeframe [6, 7, 9–51] (Fig. 1). Of the 11 organisations, only three published more than five RGs during that time period – WHO (*n* = 8), National Institute for Health and Care Excellence (NICE; *n* = 9), and the United States Centers for Disease Control and Prevention (CDC; *n* = 17). The nomenclature related to RGs was not uniform across organisations (Table 1).

**Table 1:** Nomenclature used for rapid guidelines according to institutions

Institutions	Nomenclature used to describe rapid guidelines
WHO	Rapid Advice Guideline
CDC	Interim Guidance
NICE	Short Clinical Guideline
American College of Medical Toxicology	Position Statement – Interim Guidance
Public Health Agency of Canada	Interim Guidance
Health Protection Agency (United Kingdom)	Interim United Kingdom Guidelines
*Sociedad Española de Oncología Médica* (Spanish Society of Medical Oncology)	Clinical Guidelines
WHO Global Malaria Programme	Interim Position Statement; Updated WHO Policy Recommendation
The Royal College of Ophthalmologists (United Kingdom)	Interim Guidelines

*CDC* United States Centers for Disease Control and Prevention, *NICE* National Institute for Health and Care Excellence, *WHO* World Health Organization

#### Characteristics of included guidelines

Table 2 depicts the key characteristics of the RGs evaluated. WHO guidelines (*n* = 8) were related to chronic infectious diseases including HIV and emerging infections [7, 11, 15, 22, 25, 31, 39, 47]. The reasons provided for RG were the sudden increase in incidence along with high case-fatality rates of the disease (e.g. human cases of avian influenza), the emergence of new treatment modalities, new evidence on existing treatments or new diagnostic tests. For two guidelines, HIV and infant feeding and tuberculosis in children, the rationale was not described [10, 11]. The development timeframe varied from 5 to 12 months.

**Table 2:** Characteristics of the rapid guidelines according to organisations (*n* = 36)

	WHO (*n* = 8)	NICE (*n* = 9)	CDC (*n* = 17)	Others^a^ (*n* = 12)
Number of recommendations per document^b^	11.6 (5–27)	18.5 (12–40)	6.9 (1–19)	4.1 (1–53)^c^
Timeframe (months)^b^	8.5 (5–12)	21 (15–26)	NR	NR
New systematic reviews	4	8	NR	1^d^
Quality of evidence assessment	Yes	Yes	NR	1^d^
Economic analysis	No	Yes	No	No

*CDC* United States Centers for Disease Control and Prevention, *NICE* National Institute for Health and Care Excellence, *NR* not reported, *WHO* World Health Organization

^a^ Others include: American College of Medical Toxicology, Health Protection Agency Group A Streptococcus Working Group, International Society of Ultrasound in Obstetrics and Gynecology, Public Health Agency of Canada, the Royal College of Ophthalmologists (United Kingdom), *Sociedad Española de Oncología Médica* (Spanish Society of Medical Oncology), WHO Global Malaria Programme. In addition, one guideline published on behalf of the following organisations American Academy of Allergy, Asthma & Immunology; American Academy of Pediatrics; American College of Allergy, Asthma & Immunology; Australasian Society of Clinical Immunology and Allergy; Canadian Society of Allergy and Clinical Immunology; European Academy of Allergy and Clinical Immunology; Israel Association of Allergy and Clinical Immunology; Japanese Society for Allergology; Society for Pediatric Dermatology; and World Allergy Organization

^b^ Median (range)

^c^ 1 not reported

^d^ 11 not reported

NICE Short Clinical Guidelines (*n* = 9) focused primarily on the diagnosis or treatment of chronic diseases [6, 26, 33, 34, 36–38, 49, 52]. The reasons for conducting an RG were not always clear; however, some RGs reported rationale as uncertainty in disease management or in clinical practice. The timeframe from the decision to develop a guideline to publication varied from 15 to 26 months.

CDC produced the greatest volume of RGs during the duration of this study (*n* = 17). CDC refers to them as ‘Interim Guidance’ documents. These RGs were mostly related to emerging infection and virus control and management [20, 21, 23, 24, 27–31, 40, 41, 43, 45, 46, 50–52]. The rationale for developing RGs on influenza, Zika, anthrax, and Ebola control and management was based on the uncertainty about the disease evolution and transmission, the lack of an effective vaccine, and high mortality and morbidity. The scope of the CDC RG was broad and varied from case definitions, surveillance for different settings (e.g. schools) and treatment with antivirals. The CDC also produced RGs related to other infections (e.g. osteoarticular infections, polio vaccination, HIV prevention and prophylaxis) [20, 21, 23, 24, 29]. The reasons to develop these RGs, in general, were to present new evidence and provide recommendations to reduce the risk of disease transmission and infection. References for the tools used to assess the quality of evidence were found in RGs of WHO and NICE, but not in the CDC.

The remaining 12 RGs were produced by other institutions (Table 2) [9, 12–19, 42, 44, 48]. The RG topics were very broad, related to infectious diseases, cancer or lipid control. The Public Health Agency of Canada published two RGs on avian influenza and SARS [18, 19]. The reasons to develop the guidelines were not explicit in the text or supplement materials; however, we suspected the impetus for formulating guidance was to benefit healthcare organisations and workers in the management of people with suspected avian influenza. Generally, these institutions did not assess the quality of evidence, except for the ‘Interim UK guidelines for management of close community contacts of invasive group A streptococcal disease’, which used the Scottish Intercollegiate Guideline Network approach [9, 53]. The WHO Global Malaria Programme published three RGs related to malaria, varying from larva control, drugs management and pregnancy [15–17]. There was no description of the WHO Global Malaria Programme RG timeframe, the number of questions was not clear and the reason to conduct a RG was not reported. The timeframe was 7–8 months, similar to other WHO RGs.

#### Methodological quality of included guidelines

On the AGREE II instrument, the credibility of WHO, NICE and CDC guidelines varied across domains (Table 3). Participation of stakeholders was usually described in NICE and WHO guidelines while, among guidelines from CDC and other institutions, the scores in that domain were low, varying from 6% to 69%. Similarly, regarding group composition, patient participation was identified in all NICE guidelines, in 30% of WHO guidelines, in only one CDC guideline, and was not reported in the guidelines from other institutions. NICE guidelines described public consultation. Experts in guideline methods participated in all NICE guidelines and in 30% of WHO guidelines, but other institutions did not mention their participation. The use of tables summarising the characteristics and effects across studies or frameworks occurred in most of the NICE guidelines and approximately half of the guidelines from WHO; however, there was no description of these in the guidelines from other institutions. WHO reported the methods used to achieve consensus among members of the guideline development group in the majority of their guidelines; no other organisations mentioned their decision-making processes. Within the domain ‘rigor of development’, both WHO and NICE guidelines scored higher than the CDC and others. WHO guidelines had lower scores in the applicability domain. Only WHO and NICE consistently described the external review process for their guidelines. Regarding editorial independence, WHO guidelines achieved the highest scores compared with NICE, CDC and others. In addition, AGREE II scores for clarity of presentation were high for both WHO and NICE guidelines. The median (minimum and maximum) AGREE II overall assessment score was 6.0 (5.0–6.5) for NICE, 6.0 (4.5–6.5) for WHO and 4.5 (3.5–5.0) for CDC. The overall assessment score for other institutions was 5.0 (2.5–5.0).

**Table 3:** Evaluation of the process of rapid guidelines’ development and the quality of reporting applying the AGREE II according to organisations^a^

AGREE II domain	WHO (*n* = 8)	NICE (*n* = 9)	CDC (*n* = 17)	Others^b^ (*n* = 12)
Scoping and purpose	81% (67–97%)	94% (83–97%)	69% (53–97%)	67% (22–89%)
Stakeholder involvement	72% (67–81%)	92% (86–100%)	32% (6–69%)	33% (6–67%)
Rigor of development	71% (57–84%)	91% (88–95%)	10% (0–34%)	36% (5–75%)
Clarity of presentation	92% (81–97%)	93% (89–100%)	82% (61–100%)	83% (21–97%)
Applicability	54% (44–60%)	89% (42–92%)	25% (0–44%)	21% (4–67%)
Editorial independence	83% (75–96%)	81% (63–100%)	19% (0–25%)	25% (0–67%)
Quality (1–7)	6 (5–6.5	6 (4.5–6.5)	4.5 (3.5–5)	5 (2.5–5)

*AGREE II* Appraisal of Guidelines for Research and Evaluation II, *CDC* United States Centers for Disease Control and Prevention, *NICE* National Institute for Health and Care Excellence, *WHO* World Health Organization

^a^ Figures are medians (range)

^b^ Others include: American College of Medical Toxicology, Health Protection Agency Group A Streptococcus Working Group, International Society of Ultrasound in Obstetrics and Gynecology, Public Health Agency of Canada, the Royal College of Ophthalmologists (United Kingdom), *Sociedad Española de Oncología Médica* (Spanish Society of Medical Oncology), WHO Global Malaria Programme. In addition, one guideline published on behalf of the following organisations American Academy of Allergy, Asthma & Immunology; American Academy of Pediatrics; American College of Allergy, Asthma & Immunology; Australasian Society of Clinical Immunology and Allergy; Canadian Society of Allergy and Clinical Immunology; European Academy of Allergy and Clinical Immunology; Israel Association of Allergy and Clinical Immunology; Japanese Society for Allergology; Society for Pediatric Dermatology; and World Allergy Organization

### Methods manuals

#### Search results

Of the 35 methods manuals previously identified on guideline development, only WHO, NICE and CDC provided guidance on RG development (Table 4) [1, 54, 55].

**Table 4:** Comparison of rapid guidelines methods used by WHO, NICE and CDC

Organisation	WHO	NICE	CDC
Source document (date)	Handbook for Guideline Development (2014)	Process and Methods Guide: The Guidelines Manual (2012)	Guidelines and Recommendations: A CDC Primer (2012)
Nomenclature	Rapid advice guidelines	Short clinical guidelines	Interim guidance
Definition	Evidence-informed guidelines produced within 1–3 months providing global leadership and timely guidance in response to emergencies or to an urgent need	Guidelines that address only part of a care pathway, allowing rapid development of guidance on aspects of care for which the NHS requires urgent advice	Interim guidance is developed in response to emergencies or to rapid increases in cases of a disease or condition
Timeframe (rapid guidelines)	1–3 months	11–13 months	Not reported
Timeframe (standard guidelines)	6 months to 2 years	18–24 months	Not reported
Shortcuts identified in the methods	Limiting the scope of the review, the outcomes, add more resources to have more reviewers working in parallel; streamlining the process If necessary, use methods for timely delivery of evidence synthesis	Focused scope: 3–6 review questions; topics for health economic analysis are identified during the scoping phase; shorter period for consultation process: 4 weeks	Objective documents, usually with less than three pages, not describing methodological issues Might not need to be vetted internally

*CDC* United States Centers for Disease Control and Prevention, *NICE* National Institute for Health and Care Excellence, *WHO* World Health Organization; [1, 54, 56]

#### WHO

WHO defines a rapid advice guideline as an evidence-informed guideline produced within 1–3 months providing global leadership and timely guidance in response to emergencies or to an urgent need [1]. The timeframe in which guidelines are required differentiates the planning for the development of standard or rapid advice guidelines. The methods for guideline development should follow the same basic steps as for a standard guideline with modifications as required to meet the timeline dictated by the emergency. The WHO approval and quality control processes may be accelerated for this type of guideline. For example, rapid advice guidelines may be described as evidence based, but are not necessarily supported by a standard systematic review of evidence, whereas standard guidelines are expected to be supported by standard systematic evidence reviews. Additionally, the peer-review process for rapid advice guidelines may be limited to a review of the completed draft only, immediately before final clearance. Peer review may occur just before clearance and reviewers may be limited in number to between three and six experts rather than more, and precluded in some situations based on time constraints. Standard guidelines require a more complete peer-review process. In comparison, more comprehensive peer review is required for standard guidelines, including a review of the questions, a review of the evidence tables and draft recommendations, and a record of responses to the peer review and changes made to the document. Rapid advice guidelines require a defined date and plan for updating or conversion to a standard guideline.

The WHO Handbook specifies two types of guidelines in response to an emergency or urgent need, namely ‘rapid advice guidelines’ as discussed above and ‘emergency (rapid response) guidelines’ [1]. WHO develops emergency guidelines when a public health emergency necessitates a response within hours to days; the recommendations may be based on previous guidelines or even expert opinion [1]. However, if the public health emergency continues for an extended period, emergency guidelines must be reviewed, taking into account both the evidence emerging from the current emergency and a systematic review of the relevant evidence.

#### NICE

NICE produces RGs called ‘Short Clinical Guidelines’, which address only part of a care pathway and are intended to allow for the rapid development of guidance on aspects of care for which the National Health Service requires urgent advice [56]. The Short Clinical Guidelines steps and their timeframes are presented in the NICE Guidelines Manual [56].

The development of Short Clinical Guidelines are similar to those of standard clinical guidelines; however, the timeframe is shorter and the scope much narrower [56]. The development phase takes 4–6 months, during which the Guideline Development Group meets approximately every 4–6 weeks. Only 3–6 review questions are usually considered, compared with 15–20 in the standard clinical guideline process, resulting in 5–20 recommendations. Additionally, in contrast to standard guidelines, the Short Clinical Guideline process does not involve updating searches near the end of the development process and the consultation period is only 4 weeks. However, NICE generally does incorporate an economic assessment in Short Clinical Guidelines.

#### CDC

CDC produces a large number of recommendations covering a broad range of disciplines (e.g. physical activity, treatment of influenza, motor vehicle safety, etc.). CDC guidelines address surveillance, programme implementation and policy interventions, among others. To provide development and reporting standards to improve the transparency, validity and reliability of CDC guidelines and recommendations, CDC published the ‘Guidelines and Recommendations: a CDC primer’ [54]. Taking into account the variety of audiences, topics and communications formats, this document describes the critical elements and standards relevant to CDC guidelines. CDC develops ‘Interim Guidance’ using a shortened process in response to emergencies or to a rapid increase in prevalence or incidence of a disease or condition. This Interim Guidance refers to recommendations made between a few weeks and a couple of months and we therefore included them as RGs in our reviews. Interim Guidance may also be based on tentative or emerging data (e.g. the use of face masks and respirators during an influenza pandemic) and often updated when new evidence is available. *Morbidity and Mortality Weekly Report* or other peer-reviewed journals usually publish these RGs.

## Discussion

There is a need for guidelines in public health emergencies and humanitarian crises; however, the typical time required to produce a standard guideline (2 years or more) is not appropriate in these situations. The development of RGs is an alternative to meet the needs of policy-makers, programme managers and healthcare workers. However, there is limited information about how RGs are developed and implemented and their impact on health outcomes. This study presents current practices for RG development and the quality of RGs produced by diverse organisations.

Differences between the RG organisations identified in this study include the rationale for the development of RGs to justify the shortened timeframe. WHO RG defined the rationale for developing RGs more clearly than NICE or CDC. Often, the reason was implicit, such as the threats of new emerging diseases (e.g. severe acute respiratory syndrome) and avian influenza A (H5N1) infection, as well as pandemics of chronic diseases requiring international action and recommendations [55]. Only a few of the identified guidelines reported the duration of development (ranging from 5 to 26 months) [10, 55, 57]. Additionally, few RGs reported the involvement of guideline methodologists or end-users (e.g. patients, consumers, programme managers, etc.) during development.

We found that CDC documents included fewer recommendations when compared with WHO and NICE RGs, while failing to report the use of systematic reviews and the timeframe to develop the guidance. Standard systematic reviews often take from 6 months to 1 year to complete [58]. To address requests for literature reviews in shorter time periods and to facilitate informed decision-making and understand the credibility of rapid reviews, their methods should be explicit and transparent [59]. Butler [60], studying rapid assessments, identified that selection bias, publication bias and language of publication bias may be introduced when using literature that is readily accessible to a researcher. Ganann et al. [58] noted that rapid reviews with shorter timeframes (1–3 months) were often less systematic in their search for evidence than those with longer timeframes (3–6 months). Another study by Watt et al. [61] showed that full reviews were more likely than rapid reviews to report clinical outcomes, economic factors and social issues, and to provide greater depth of information and detail in recommendations. The authors suggested that using rapid reviews might lead to uncertainty around the conclusions drawn and inability to answer certain types of questions (e.g. economic analyses). However, they found that, although the scope of rapid reviews is limited, they can provide adequate advice for clinical and policy decisions. For RG developers, considering the amount of resources required to maintain quality in a short timeframe remains a challenge but can be overcome by working in larger and qualified teams [59, 60]. In fact, remaining systematic in the identification and use of evidence is equally important for RGs as it is for standard guidelines and RGs should not rely on expert opinion without stating the evidence from which this opinion is formed.

We found that timeframes were inconsistent with the organisations’ methods manuals. While WHO recommends RGs be developed in a timeframe between 1 and 3 months, the median time required based on our systematic survey was 8.5 months. For the NICE guidelines, the results showed a median time of 21 months while the manual suggests a timeframe of between 11 and 13 months. We identify some reasons why the proposed timeframe is not followed by the RG developers.

One point would be that RG developers were using the standard guidelines methods in a shortened period. If so, even a streamline process is not enough to fit their timeframe. Another option would be that they were using shortened methods for RG development. Again, it seems that the approach does not match the definition of RGs according to their methods manuals. Therefore, it seems imperative to define the minimal parameters to define RGs and propose their developmental methods accordingly. Otherwise, the lack of standardised RG development may lead to widely varying recommendations.

The organisations stated in their manuals that the rigorous adherence to the systematic use of evidence is the basis for all policies and that the methods applied for standard guidelines should be followed by the RGs even when under time pressure constraints [1, 54, 56]. Woolf et al. [62] advocated that the analytic framework of a guideline is a key element in its development. It is in this critical stage that a group defines which questions must be answered to arrive at a recommendation. However, in the majority of the RGs assessed in this systematic survey, many details were not identified, including how the evidence was assessed, the timeframe needed to conduct the process, the number of key questions for the RGs, and the procedures for editorial independence and external review. The WHO and NICE RGs described the use of systematic reviews and the assessment of the quality of evidence; however, there was no information regarding the process of consensus [1, 7]. Declaration of interest and conflict of interest management were also not described in the majority of the RGs in the present study, in line with other findings in the literature [63]. One possibility is that the steps were conducted but not reported.

Although RGs may be designed to reduce the work and time necessary to complete a guideline, they should provide the same information as a standard guideline, explicitly indicating the methods used and recognition of the potential bias introduced by the abbreviated methods [58, 64]. This information should be clearly and transparently described enabling end-users to balance the certainty placed in recommendations and the evidence underlying them [55]. For example, describing that systematic reviews were not used in the process because there was none available in the period that the guideline was developed is informative, instead of simply omitting the information.

In the present review, no institution reported using a pilot study or other mechanism for guideline implementation. Gagliardi and Brouwers [65] observed that there was a lack of details regarding guideline implementation in the literature and that new approaches for guideline development and implementation may need to be developed to enhance the use of guidelines. Shekelle et al. [66] suggest that identification of potential barriers to the implementation of recommendations and strategies for guideline dissemination should be addressed in the guidelines. WHO emphasised that the RGs should be developed only for situations where dissemination and implementation would be feasible when considering the health systems, acceptability of the intervention, training and resources available [1]. While RGs may be developed in a transparent manner and with methodological rigor, the implementation should also be evaluated to assess the impact of RGs.

Our review of the credibility of RGs identified variation between documents and across organisations. While documents from WHO rated highest when evaluated with AGREE II, our results suggest that organisations should plan their guidelines according to tools such as the GIN-McMaster Checklist to achieve high ratings on the AGREE II instrument [5]. The third article in this series provides guidance for applying the checklist to RGs [4]. Two domains of importance include transparency in the involvement of external stakeholders, including patient participation and the process for public consultation.

The strengths of this study include the extensive and systematic literature review, aggregating electronic, experts’ opinions and hand searching (Additional file 1: Appendix 4). Further, the application of piloted and standardised methods to extract data conducted in pairs contributed to the robustness of the analysis. The weaknesses of this study include the exclusion of methods manuals and guidelines not published in English, as well as reports or documents that used other nomenclature that may have been developed following a shortened timeframe for their development. There are a wide variety of terms and definitions used among the organisations for RGs, which made it difficult to identify them and assess their methodology and characteristics. Finally, we do not know how the AGREE II scores of RGs compare to guidelines developed without time constraints but suggest that this could be further explored.

We did identify one example of guidelines developed within a shortened timeframe whilst maintaining high quality methods [55]. In response to the avian influenza pandemic (H5N1), WHO developed a RG within 4 months using the Grading of Recommendations Assessment, Development and Evaluation (GRADE) approach. When assessed using AGREE II, this guideline was determined to be of high quality. Although one of the authors of the current article was an author of that guideline, the assessment of the guideline using AGREE II was conducted by authors not involved in developing that guideline. Preceding uptake of rapid guideline development in the WHO handbook, the authors of the guidelines explicitly reported the methods used. The authors attributed the transparency and the short amount of time used to prepare the guideline (4 months) to utilising the GRADE approach and methods developed for the WHO shortly before the guideline work began [55]. They suggested that the time can be reduced by identifying collaborating centres capable of elaborating evidence profiles and also by building up in-house capacity to reduce the time needed to organise a review team. The use of tools such as the GRADEpro Guideline Development tool (www.gradepro.org) that keep records of evidence, decisions and judgments, facilitates the development of updated RGs, as recently demonstrated in WHO delamanide and bedaquiline guidelines for multi-drug resistant tuberculosis [67].

## Conclusions

There is a lack of standardised nomenclature, definitions and processes regarding RG development. Only three institutions (WHO, NICE and CDC) were identified as routinely developing RGs. However, the reasons for developing RGs were not always clear and varied widely. We identified inconsistencies in the proposed methods and non-adherence to self-imposed timeframes. The methods used to assess the quality of evidence, the consensus process and the management of the conflicts of interests were not always transparent. However, we also identified a RG that achieved its goal of maintaining credibility if there is a concentration of skilled resources [55]. Organisations developing RGs can provide important service by using a robust and transparent process that simplifies adaptation of RGs to specific settings.

## Additional file

Additional file 1: **Appendix 1.** Protocol. **Appendix 2.** Search strategies. **Appendix 3.** List of predefined societies for manual search. **Appendix 4.** PRISMA Checklist. (DOCX 627 kb)

## Abbreviations

AGREE II: Appraisal of Guidelines for Research & Evaluation Instrument
CDC: United States Centers for Disease Control and Prevention
GRADE: Grading of Recommendations Assessment, Development and Evaluation
NICE: National Institute for Health and Care Excellence
RG: rapid guideline
