# Supplementary material for: Development of rapid guidelines: 1. Systematic survey of current practices and methods
Source: Health Res Policy Syst. 2018 Jul 13;16:61. doi: 10.1186/s12961-018-0327-8 (PMC6044042; doi:10.1186/s12961-018-0327-8)

# Additional file 1

# Appendix 1. Protocol

**Systematic Review of Rapid Guidelines and Methods for its Development [Protocol]**

**Background**

Guidelines are recommendations intended to assist providers and recipients of health care and other stakeholders to make informed decisions ([1](#_ENREF_1)). Thus, guidelines should include a clear recommendation for action, based not only in the best available evidence for effects of interventions on health outcomes, but also factors such as resource allocations, values and preferences. However, integrating these various factors in recommendations, makes the processes complex and time consuming.([2](#_ENREF_2), [3](#_ENREF_3))

Time involved is a main issue in developing guidelines; often a guideline is published two or more years after its conception.([4](#_ENREF_4), [5](#_ENREF_5)) The average time taken to develop a standard SIGN guideline is 28 months;([6](#_ENREF_6)) NICE estimate its process in 18 to 24 months.([7](#_ENREF_7)) This timeframe is not practical for providing guidance in situations when rapid advices are needed, for example for emerging infectious diseases such as avian influenza or severe acute respiratory syndrome;([4](#_ENREF_4)) for these situations, guidance produced in a shorter period is needed. Furthermore, despite limited timelines, it is important that rapid guidelines remain credible.

The objective of this work is to describe current practices about the development of rapid guidelines, emphasizing on the key concepts underlying its development. We will conduct a systematic review of the existent literature in order to identify the factors that stimulate the development of rapid advice guidelines instead of standard guidelines, to identify methodological approaches that allow quicker development of recommendations, and to evaluate the quality of published rapid guidelines using the AGREE-II instrument.([8](#_ENREF_8)) In addition, we aim to assess the implications of taking methodological shortcuts from standard guidelines in terms of biased recommendations (Table 1).

**Methods**

This systematic review is part of the support material to be prepared for a WHO project aiming at the development of a process and standards for WHO rapid advice guidelines.

In this systematic review, we are defining “rapid guidelines” as those guidelines produced with a shortened timeline compared to standard guidelines produced by the same organization or labeled by an organization as “rapid”.

*Eligibility criteria*

We will include the following sources of literature:

1. Methodological manuals and publications addressing the issue of developing guidelines using a shortened framework.
2. Guidelines described as “rapid”, using a shortened framework for its development.

We will not include guideline updates, rapid systematic reviews and rapid health technology assessment as well as guidelines not providing healthcare recommendations (e.g. guidelines about laboratory procedures). Guidelines not published in English will be excluded.

*Search strategy and study selection*

We will perform an iterative search strategy. First, an exploratory electronic search will be performed in the following search engines: Trip Database, PubMed, Embase and National Guideline Clearinghouse. (Appendix 1) The main purpose of this search is to identify organizations that are developing guidelines with a rapid framework.

Second, we will contact experts from key organizations (e.g. guidelines developers, methodologists) in order to identify methodological manuals and publications that describe the process of guideline development and to identify potential sources of rapid guidelines (e.g. societies’ websites). We will stop the process when we reach saturation, (i.e. no new manuals or publications are identified through expert contact).

Third, based on the findings from the first two steps, an experienced librarian and a clinical epidemiologist will develop a search strategy in order to identify rapid guidelines. From the electronic search, all citations identified will be entered into an electronic database. Initially, two investigators will independently screen potentially relevant studies through the titles and abstracts; when needed, a full text will be obtained to determine the article's eligibility. The manual search will also be performed by two investigators independently. Discrepancies, both in electronic and manual search, will be solved by discussion until reach consensus. All processes will be piloted.

Fourth, we will perform a manual search of selected sources in order to identify methodological manuals and rapid guidelines (see appendix 2); in addition, a manual search will be performed of all suggested sources and reference lists of included documents will be evaluated.

*Data management*

Experts’ suggestions of sources of information will be presented, as well as respondents’ profile and response rate. Data will be presented separately for rapid advice guidelines and for methodological manuals.

Rapid advice guidelines

Two independent investigators will review the eligible studies and extract data using a standardized and pilot tested form. For our scoping exercise, four investigators abstracted data from two potential rapid guidelines identified.([9](#_ENREF_9), [10](#_ENREF_10)) For the systematic review we will abstract the following data: (1) condition evaluated; (2) guideline group composition, (3) number of questions framed; (4) number of recommendations; (5) guideline’s timeline (including time spent in each step and time to completion), (6) evidence review process, (7) quality of evidence assessment procedures, (8) factors considered when moving from the evidence to recommendations, (9) considerations about costs and stakeholder involvement and (10) reasons for proceeding the development as a rapid guideline instead of a standard guideline. Also, to assess the methodological quality, two investigators will apply the AGREE-II instrument to all rapid guidelines identified. Discrepancies will be solved by discussion.

Methodological manuals

From the methodological manuals, two investigators will abstract data regarding the specific development of rapid guidelines using a standardized form, including the key concepts for its development and the main differences when compared to standard guidelines. We will compare and contrast the guidance provided by the identified organizations on how to produce rapid guidelines. Findings will be presented narratively in a group meeting for drafting the results.

**Discussion**

*Expected significance of the study*

This systematic review is the first step in a project supporting WHO in developing a process and standards for WHO and other organizations’ rapid advice guidelines.

We aim to review the current status of development of rapid guidelines, helping guideline developers in the process of developing clinical guidelines when a shortened timeline is needed. Also, we aim to identify gaps in the knowledge about this issue, that will be researched in a further study employing a qualitative approach.

**References**

1. World Health Organization. WHO Handbook for guideline development: World Health Organization; 2014.

2. Guyatt GH, Oxman AD, Kunz R, Falck-Ytter Y, Vist GE, Liberati A, et al. Going from evidence to recommendations. BMJ. 2008;336(7652):1049-51.

3. Oxman A, Fretheim A, Schunemann H, SURE. Improving the use of research evidence in guideline development: introduction. Health Research Policy and Systems. 2006;4(1):12.

4. Schunemann HJ, Hill SR, Kakad M, Vist GE, Bellamy R, Stockman L, et al. Transparent development of the WHO rapid advice guidelines. PLoS Med. 2007;4(5):e119.

5. Raine R, Sanderson C, Black N. Developing clinical guidelines: a challenge to current methods. BMJ. 2005;331(7517):631-3.

6. Scottish Intercollegiate Guidelines Network. SIGN 50: A guideline developer’s handbook. Edinburgh: Scottish Intercollegiate Guidelines Network, 2011.

7. National Institute for Health and Clinical Excellence. The guidelines manual. London: National Institute for Health adn Clinical Excellence, 2012.

8. Brouwers MC, Kho ME, Browman GP, Burgers JS, Cluzeau F, Feder G, et al. AGREE II: advancing guideline development, reporting and evaluation in health care. Canadian Medical Association Journal. 2010;182(18):E839-E42.

9. National Institute for Health and Care Excellence. Osteoporosis: assessing the risk of fragility fracture. National Institute for Health and Care Excellence, 2012.

10. World Health Organization. Rapid advice: Diagnosis, prevention and management of cryptococcal disease in HIV-infected adults, adolescents and children. Geneva: World Health Organization, 2011.

Table 1 – Study’s objectives

- To quantify the existent literature about the issue: rapid guidelines produced and methodological manuals addressing its development
- To identify the factors that stimulate the development of rapid advice guidelines instead of standard guidelines,
- To identify methodological shortcuts that allow quicker development of recommendations in guidelines
- To evaluate the quality of published rapid guidelines according to the AGREE-II instrument.
- To assess the implications of taking methodological shortcuts from standard guidelines in terms of biased recommendations.

Appendix 1: Search strategy

*“rapid, fast, short, interim”*

Appendix 2: List of pre-defined societies’ websites for manual search:

- World Health Organization (WHO)
- National Institute of Clinical Excellence (NICE)
- Scottish Intercollegiate Guidelines Network (SIGN)
- National Health and Medical Research Council (NHMRC)
- U.S. Preventive Services Task Force (USPSTF)
- Canadian Task Force on Preventive Health Care (CTFPHC)
- Public Health Agency of Canada (PHAC)
- Centers for Disease Control and Prevention (CDC)
- European Centre for Disease Prevention and Control (ECDC)

# Appendix 2. Search strategies

**(ORIGINAL)** All searches up to March 2013.

**TRIP database**

title: rapid OR quick OR interim OR short OR fast OR practical; and restrict to guidelines

For the TRIP search http://www.tripdatabase.com/search?categoryid=&criteria=%28title%3Arapid+OR+quick+OR+interim+OR+short+OR+fast%29. (91)

**Embase 1996 to 2013**

1 exp practice guideline/     274961

2 (guideline* or guidance or recommendation* or statement).m_titl.   81542

3 ((rapid or quick or interim or fast or short) adj3 (process or method or methods or approach or guidance or guideline or guidelines)).tw.  40891

4 1 and 2 and 3    228

**Ovid MEDLINE(R) In-Process & Other Non-Indexed Citations and Ovid MEDLINE(R) <1946 to 14 march 2013>**

1     ((rapid or quick or interim or fast or short or practical) adj3 (process or method or methods or approach or

guidance or guideline or guidelines)).tw. (63583)

2     practice guideline.pt. (17519)

3     1 and 2 (186)

**Updated search strategy – December 31, 2014**

using the same strategies addressed above for MEDLINE and EMBASE and limiting the publication time from the original search day until December 2014

Database: Embase <1996 to 2014 Week 50>, Ovid MEDLINE(R) In-Process & Other Non-Indexed Citations and Ovid MEDLINE(R) <1946 to Present>

Search Strategy:

1     exp practice guideline/ (332233)

2     (guideline* or guidance or recommendation* or statement).m_titl. (193740)

3     ((rapid or quick or interim or fast or short) adj3 (process or method or methods or approach or guidance or guideline or guidelines)).tw. (105341)

4     1 and 2 and 3 (298)

5     ((rapid or quick or interim or fast or short or practical) adj3 (process or method or methods or approach or guidance or guideline or guidelines)).tw. (136263)

6     practice guideline.pt. (20171)

7     5 and 6 (240)

8     4 or 7 (495)

9     limit 8 to yr="2013 -Current" (69)

10     remove duplicates from 9 (62)

**Updated search strategy – March 21, 2018**

**TRIP database**

title: rapid OR quick OR interim OR short OR fast OR practical (71,253)

restrict to guidelines (1,476)

Limit to 2013 to current (502)

**Updated search strategy – March 21, 2018**

using the same strategies addressed above for MEDLINE and EMBASE and limiting the publication time from the original search day until December 2014

Database: Embase <1996 to 2014 Week 50>, Ovid MEDLINE(R) In-Process & Other Non-Indexed Citations and Ovid MEDLINE(R) <1946 to Present>

Search Strategy:

1     exp practice guideline/ (467918)

2   (guideline* or guidance or recommendation* or statement).m_titl. (277582)

3   ((rapid or quick or interim or fast or short) adj3 (process or method or methods or approach or guidance or guideline or guidelines)).tw. (150984)

4     1 and 2 and 3 (391)

5   ((rapid or quick or interim or fast or short or practical) adj3 (process or method or methods or approach or guidance or guideline or guidelines)).tw. (196179)

6     practice guideline.pt. (23711)

7     5 and 6 (302)

8     4 or 7 (648)

9     limit 8 to yr="2013 -Current" (221)

10   remove duplicates from 9 (210)

# Appendix 3. List of predefined societies for manual search

- Agency for Healthcare Research and Quality (AHRQ)
- World Health Organization (WHO)
- National Institute for Health and Care Excellence (NICE)
- Scottish Intercollegiate Guidelines Network (SIGN)
- National Health and Medical Research Council (NHMRC)
- U.S. Preventive Services Task Force (USPSTF)
- Canadian Task Force on Preventive Health Care (CTFPHC)
- Public Health Agency of Canada (PHAC)
- Centers for Disease Control and Prevention (CDC)
- European Centre for Disease Prevention and Control (ECDC)

# Appendix 4. PRISMA Checklist


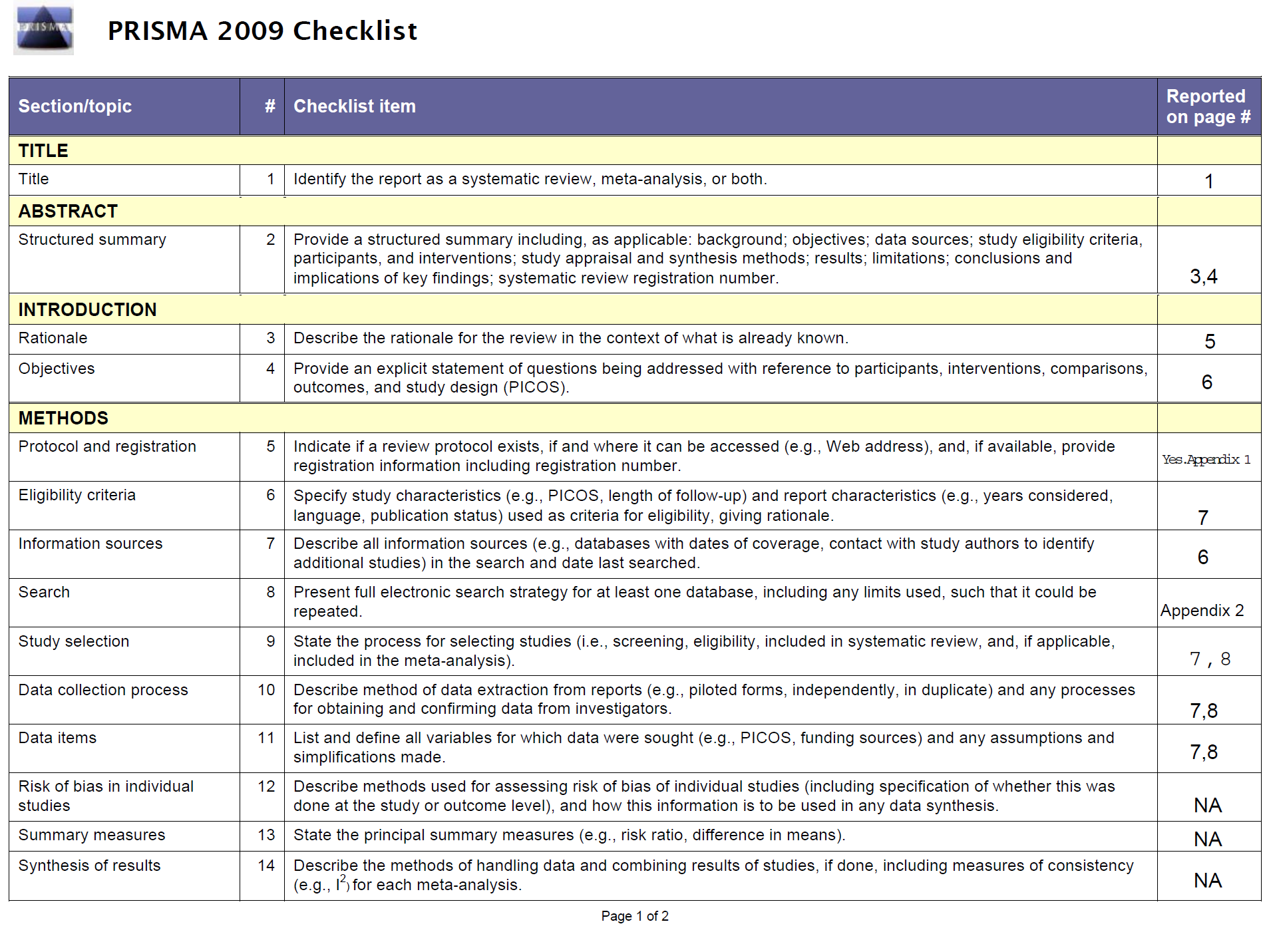


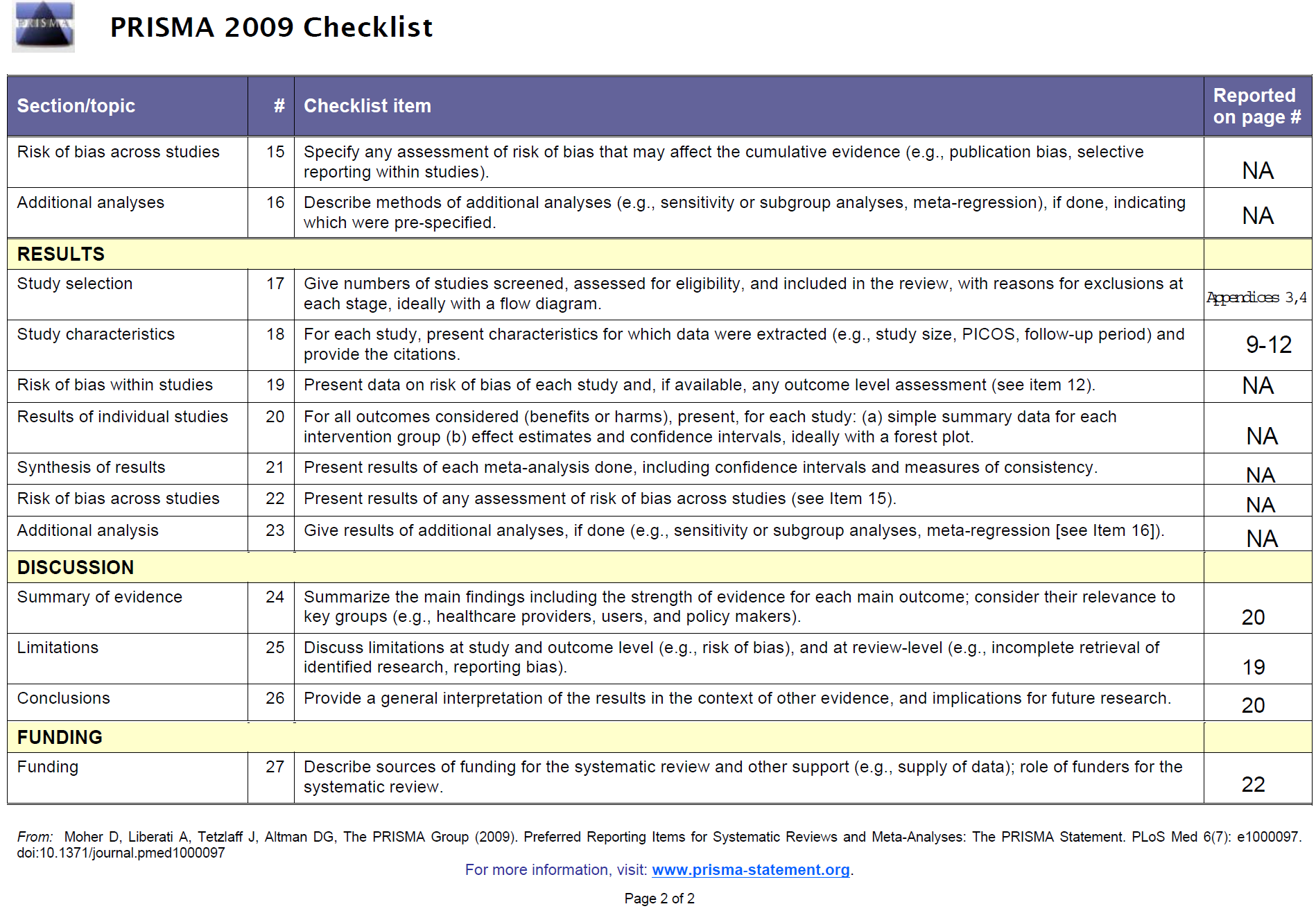

Supplement: Supplementary file 1 — Appendix 1. Protocol. Appendix 2. Search strategies. Appendix 3. List of predefined societies for manual search. Appendix 4. PRISMA Checklist. (DOCX 627 kb) [file 12961_2018_327_MOESM1_ESM.docx]
